# The influence of antibiotic administration on the outcomes of head-and-neck squamous cell carcinoma patients undergoing definitive (chemo)radiation

**DOI:** 10.1007/s00405-023-07868-3

**Published:** 2023-02-10

**Authors:** Alexander Rühle, Jiadai Zou, Margaretha Glaser, Lennard Halle, Eleni Gkika, Henning Schäfer, Andreas Knopf, Christoph Becker, Anca-Ligia Grosu, Ilinca Popp, Nils H. Nicolay

**Affiliations:** 1 Department of Radiation Oncology, University of Freiburg–Medical Center, Robert-Koch-Str. 3, 79106 Freiburg, Germany; 2 German Cancer Consortium (DKTK), Partner Site Freiburg, German Cancer Research Center (DKFZ), Heidelberg, Germany; 3 Department of Otorhinolaryngology, University of Freiburg–Medical Center, Freiburg, Germany

**Keywords:** Head-and-neck cancer, Head-and-neck squamous cell carcinoma, Radiotherapy, Chemoradiation, Antibiotics

## Abstract

**Purpose:**

Effects of antibiotic administration on patients’ microbiome may negatively influence cancer outcomes, and adverse prognoses after antibiotic application have been demonstrated for cancer patients receiving immune checkpoint inhibitors. While the microbiome may play an important role also in head-and-neck squamous cell carcinoma (HNSCC), the prognostic value of antibiotic treatment here is largely unknown. We therefore analyzed whether antibiotic prescription is associated with impaired oncological outcomes of HNSCC patients undergoing definitive (chemo)radiation.

**Methods:**

A cohort of 220 HNSCC patients undergoing definitive (chemo)radiation between 2010 and 2019 was analyzed. The influence of antibiotic administration on locoregional control, progression-free survival (PFS) and overall survival (OS) was determined using Kaplan–Meier and Cox analyses.

**Results:**

A total of 154 patients were treated with antibiotics within 30 days before (chemo)radiation (pretherapeutic) or during (chemo)radiation (peritherapeutic). While antibiotic prescription was not associated with age, ECOG, tumor localization or radiotherapy characteristics, patients treated with antibiotics had significantly higher tumor stages. Peritherapeutic antibiotic administration diminished PFS (HR = 1.397, *p* < 0.05, log-rank test) and OS (HR = 1.407, *p* < 0.05), whereas pretherapeutic administration did not. Antibiotic application was an independent prognosticator for OS (HR = 1.703, *p* < 0.05) and PFS (HR = 1.550, *p* < 0.05) in the multivariate Cox analysis within the subgroup of patients aged < 75 years.

**Conclusion:**

Peritherapeutic antibiotic usage was associated with impaired oncological outcomes in HNSCC patients undergoing (chemo)radiation. Further studies including microbiome analyses are required to elucidate underlying mechanisms.

**Supplementary Information:**

The online version contains supplementary material available at 10.1007/s00405-023-07868-3.

## Introduction

Head-and-neck squamous cell carcinoma (HNSCC) is a frequent malignancy, accounting for more than 450,000 deaths per year globally [1]. Improvements in diagnostics and therapy have resulted in improvements of patients’ outcomes over the last decades; however, outcomes for HNSCC patients remain unsatisfactory with 5-year survival rates ranging between 33% (hypopharyngeal cancer) and 70% (laryngeal cancer) [2]. (Chemo)radiation is a main treatment modality for locally advanced HNSCCs, either as a definitive or as an adjuvant treatment [3–6]. Several tumor- and patient-related parameters such as tumoral human papillomavirus (HPV) status, tumor-associated hypoxia, levels of tumor-infiltrating lymphocytes, comorbidity burden and performance status are known to influence the outcome of HNSCC patients undergoing (chemo)radiation [7–10].

Recent studied highlighted a potential role of the patients’ microbiome on the efficacy of different anti-cancer treatments [11, 12]. Especially the efficacy and toxicity profile of immune checkpoint inhibitors have been shown to depend on the patients’ gut microbiome [13]. There is also a rising number of studies reporting potential interactions between the microbiome and radiotherapy [14, 15]. For instance, germ-free mice have been demonstrated to exhibit a more radioresistant intestinal mucosa, and therapeutic manipulations of the microbiome, e.g., by fecal microbiome transplantation or administration of distinct bacteria, influenced radiotherapy-related toxicities [16, 17]. As antibiotics considerably influence patients’ gut microbiome, they may also impact the potential influence of the microbiome on the efficacy of radiotherapy [18–20]. Indeed, Nenclares and colleagues could demonstrate that peritherapeutic antibiotic administration in HNSCC patients undergoing radiotherapy may be an independent prognosticator for reduced disease-free survival [11]. However, this finding was derived from a single-center analysis and has so far not been verified by other groups. Furthermore, it has to be mentioned that other preclinical analyses observed increased anti-tumor effects of radiotherapy after vancomycin, selectively targeting gram-positive bacteria, suggesting a complex interplay of radiotherapy, patient microbiomes and antibiotic administration [21]. To make the association between radiotherapy and antibiotic administration even more complex, antibiotics such as doxycycline and cephalosporins have found to exhibit radiosensitizing abilities in vitro [22, 23].

In this analysis, we therefore analyzed whether pretherapeutic and/or peritherapeutic antibiotic prescription may have influenced oncological outcomes of HNSCC patients undergoing definitive (chemo)radiation. As both immunosenescence (i.e., the process of the immune system’s deterioration associated with increasing age) and age-related dysbiosis of the patients’ microbiome may alter the immune system’s anti-tumor abilities and the interaction between antibiotics and tumor control after radiotherapy, we also aimed to evaluate possible response differences between the cohort of the so-called older/oldest olds (≥ 75 years) and the remaining patients [24].

## Methods

### Patients and treatment

Patients treated with definitive (chemo)radiation between 2010 and 2019 at the Department of Radiation Oncology, Medical Center – University of Freiburg were analyzed regarding the impact of antibiotic prescription on the oncological outcomes. The retrospective analysis was approved by the institutional review committee of the University of Freiburg (reference no. 389/19). Patient and treatment characteristics were extracted from the electronic patient records. The 7th UICC (Union for International Cancer Control) classification was used for TNM staging.

In general, treatment decisions were based on multidisciplinary tumor board recommendations. In order to ensure exact positioning during treatment, patients were immobilized with individually moulded thermoplastic masks. Patients with locally advanced HNSCCs underwent definitive chemoradiation with doses of 50–54 Gy (EQD2, *α*/*ß* = 10) to the low-risk planning target volume (PTV) and 70 Gy (EQD2, *α*/*ß* = 10), delivered either as sequential or simultaneous boost, to the high-risk PTV. Usually, three cycles of high-dose cisplatin (100 mg/m^2^ body surface area in weeks 1, 4 and 7) were administered simultaneously during treatment in case of locally advanced HNSCCs. Patients with locally limited HNSCCs (T1–2 N0) or patients who were unable to receive concomitant chemotherapy were treated with radiotherapy alone. Three patients received concomitant cetuximab due to contraindications against cisplatin. Within the first two years after treatment completion, patients were followed-up every three months by computed tomography investigation and clinical assessment. For the third year after treatment, follow-up intervals were extended to six months, and for year 4–5, patients were followed-up annually.

### Antibiotic treatment

Information about antibiotic application was collected using the electronic patient records. Treatment was separately analyzed regarding pretherapeutic (30 days before (chemo)radiation until beginning of (chemo)radiation) and peritherapeutic antibiotic application (during (chemo)radiation). The indication for antibiotic treatment as well as the duration of administration and the applied classes of antibiotics were analyzed. While indication and classes of antibiotics referred to each course of antibiotic therapy, duration of antibiotic administration was computed per patient.

### Statistical analyses

Patient data as well as tumor and treatment characteristics were presented as median values including interquartile ranges or frequencies depending on the type of variable. The distribution of the parameters in dependence of antibiotic therapy was compared using *t* tests and *χ*^2^-test. Overall survival (OS) was calculated from the start of (chemo)radiation until death, and progression-free survival (PFS) was assessed from the beginning of (chemo)radiation until death, local/locoregional progression or distant progression. Locoregional control (LRC) was calculated from the start of treatment until first detection of a local recurrence or regional lymph node recurrence/progression. Patients were censored at the date of last follow-up consultation. Missing survival data were obtained through the tumor registry of the Comprehensive Cancer Center Freiburg (CCCF). The median follow-up time was calculated using the reverse Kaplan–Meier method. Log-rank tests were used to compare the survival rates depending on antibiotic application. Cox proportional hazards regression analyses were performed for OS and PFS, and hazard ratios (HR) with the corresponding 95% confidence intervals (95% CI) were calculated. Parameters with a *p* value < 0.1 in the univariate Cox analysis were included in the multivariate analysis (enter method). *P* < 0.05 was considered statistically significant throughout the study. SPSS Statistics software version 25 (IBM, Armonk, NY, USA) was used for statistical analyses.

## Results

### Patients and antibiotic administration

Characteristics of the study population and details of antibiotic administration are presented in Tables 1 and 2, respectively. Overall, 154 of 220 patients (70.0%) received antibiotics in the time interval ranging between 30 days before (chemo)radiation until treatment completion. A total of 100 patients were treated during the course of (chemo)radiation (i.e., peritherapeutic application), whereas 93 patients received antibiotics prior to (chemo)radiation (i.e., pretherapeutic application). Antibiotic therapy was applied mostly as perioperative single shot prophylaxis prior to port-catheter implantation (*n* = 134). Of these, the majority (*n* = 86) had their port-catheter implantation prior to the start of (chemo)radiation. Other common indications were port-catheter infections (*n* = 22), respiratory tract infections (*n* = 17), sepsis (*n* = 9) or urogenital tract infections (*n* = 8). In 30 cases, antibiotics were prescribed empirically (e.g., due to fever of unknown origin). Cephalosporins (*n* = 148), penicillins with beta-lactamase-inhibitors (*n* = 36), clindamycin (*n* = 14) and fluoroquinolones (*n* = 8) were the most commonly prescribed classes of antibiotics. The duration of antibiotic application ranged from 1 to 49 days. Median total duration of antibiotic treatment was 3 days for patients who were treated with antibiotics during (chemo)radiation, while it was 1 day for the total cohort. After excluding patients who only received perioperative single shot prophylaxis, median total duration of antibiotic treatment amounted to 11 days. Most patients received only one type of antibiotics (*n* = 108, 70.1%), 29 (18.8%) patients received 2 types, and 17 (11.3%) patients were treated with 3 or more types. A total of 227 antibiotic therapy courses were prescribed in the analyzed time interval. Of these, 209 (92.1%) were performed with intravenous antibiotics, and 18 (7.9%) with oral antibiotics.

**Table 1:** Patient and treatment characteristics for the study population according to antibiotic administration (*n* = 220)

	Overall	No antibiotics	Antibiotics	*p*
	(*n* = 220)	(*n* = 66)	(*n* = 154)	
Age, years				
Median (IQR)	69 (62–76)	72 (67–79)	67 (59–73)	0.245^a^
Gender				0.013^b^
Female	58 (26.4)	10 (15.2)	48 (31.2)	
Male	162 (73.6)	56 (84.8)	106 (68.8)	
ECOG				0.146^b^
0	130 (59.1)	36 (54.5)	94 (61.0)	
1	77 (35.0)	23 (34.8)	54 (35.1)	
2	13 (5.9)	7 (10.6)	6 (3.9)	
T stage				< 0.001^b^
1	15 (6.8)	13 (19.7)	2 (1.3)	
2	41 (18.6)	15 (22.7)	26 (16.9)	
3	67 (30.4)	12 (18.2)	55 (35.7)	
4	97 (44.1)	26 (39.4)	71 (48.1)	
N stage				< 0.001^b^
0	44 (20)	25 (37.9)	19 (12.3)	
1	15 (6.8)	6 (9.1)	9 (5.8)	
2	149 (67.7)	33 (50.0)	116 (75.3)	
3	12 (5.5)	2 (3.0)	10 (6.5)	
UICC				< 0.001^b^
I	12 (5.4)	12 (18.2)	0 (0)	
II	8 (3.6)	3 (4.5)	5 (3.2)	
III	24 (10.9)	8 (12.1)	16 (10.4)	
IV	176 (80.0)	43 (65.2)	133 (86.4)	
Tumor localization				0.490^b^
Nasopharynx	15 (6.8)	4 (6.1)	11 (7.1)	
Oropharynx	95 (43.2)	28 (42.4)	67 (43.5)	
Hypopharynx	42 (19.1)	14 (21.2)	28 (18.2)	
Oral cavity	17 (7.7)	7 (10.6)	10 (6.5)	
Larynx	34 (15.4)	9 (13.6)	25 (16.2)	
Multilevel	10 (4.5)	3 (4.5)	7 (4.5)	
Parotid gland	6 (2.7)	0 (0)	6 (3.9)	
Other salivary glands	1 (0.4)	1 (1.5)	0 (0)	
Systemic treatment				< 0.001^b^
Radiotherapy alone	41 (18.6)	28 (42.4)	13 (8.4)	
Concomitant systemic treatment	179 (81.4)	38 (57.6)	141 (91.6)	
Radiotherapy dose, Gy				
Median (IQR)	70.0 (69.3–70)	70.0 (69.3–70)	70.0 (66.0–70.0)	0.500^a^
Radiotherapy duration, days				
Median (IQR)	49 (46–53)	49 (45–52)	49 (46–53)	0.694^a^
Radiotherapy compliance				0.571^b^
Radiotherapy completed	196 (89.1%)	60 (90.9%)	136 (88.3%)	
Radiotherapy not completed	24 (10.9%)	6 (9.1%)	18 (11.7%)	

The 7th UICC TNM classification was used. Groups were compared using *t* tests or *χ*^2^-tests

*ECOG* Eastern Cooperative Oncology Group, *IQR* interquartile range

^a^*t* test

^b^*χ*^2^ test

**Table 2:** Treatment details regarding antibiotic application including duration, indication and types of antibiotics (*n* = 154 patients)

Duration of antibiotic administration	
1–3 days	97
4–7 days	10
8–21 days	39
> 21 days	8
Indication	
Port-catheter system placement	134
Empirical treatment	30
Respiratory tract infection	22
Port-catheter infection	17
Sepsis	9
Urogenital tract infection	8
Others	7
Classes of antibiotics	
Cephalosporins	148
Penicillins with beta-lactamase-inhibitors	36
Clindamycin	14
Fluoroquinolones	8
Carbapenem	6
Glycopeptides	3
Macrolide	3
Others^a^	9
Application in relation to radiotherapy	
During radiotherapy (peritherapeutic)	100
Prior to radiotherapy (pretherapeutic)	93
Applied types of antibiotics	
1	108
2	29
≥ 3	17

The data regarding indication and classes of antibiotics refer to each course of antibiotic therapy (*n* = 227). Duration of antibiotic administration summarizes the duration of all courses of antibiotic therapy per patient

^a^Linezolide (*n* = 3), rifampicin (*n* = 2), nitroimidazole (*n* = 2), nitrofurantoin (*n* = 1), doxycycline (*n* = 1)

### Antibiotic administration is not related to age, ECOG status or duration of the radiotherapy course

The necessity for antibiotic treatment was not associated with age (*p* = 0.245) or ECOG status (*p* = 0.146). Female patients were more frequently treated with antibiotics than male patients (*p* = 0.013). Furthermore, patients treated with antibiotics had significantly higher T stages, N stages as well as UICC stages (*p* < 0.001), and therefore more frequently received concomitant chemotherapy (*p* < 0.001). However, neither radiotherapy dose (*p* = 0.500) nor the overall duration of radiotherapy (*p* = 0.694) differed in dependence of antibiotic treatment. Furthermore, the proportion of patients completing the prescribed course of radiotherapy was similar between both cohorts (90.9% in the group receiving no antibiotics versus 88.3% in the group treated with antibiotics, *p* = 0.571).

### Antibiotic administration during (chemo)radiation is associated with reduced survival

**Fig. 1:**
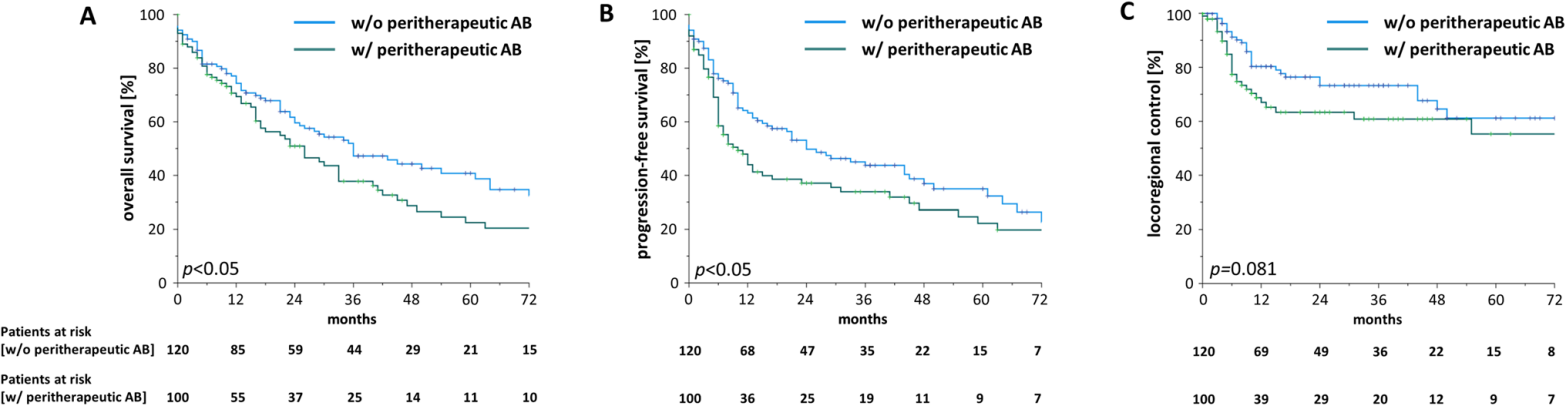
Peritherapeutic antibiotic administration impairs oncological outcomes in HNSCC patients undergoing radiotherapy. Overall survival (**A**), progression-free survival (**B**) and locoregional control (**C**) of HNSCC patients undergoing definitive radiotherapy (either alone or with concomitant systemic treatment) depending on peritherapeutic antibiotic administration. Groups were compared using log-rank tests. *AB* antibiotics, *w*/ with, *w*/*o* without

After a median follow-up time of 50 months, the median OS ranged at 30 months. During the follow-up time, 129 patients (58.6%) died, and 58 (26.4%) developed local or locoregional recurrences. Kaplan–Meier estimates for LRC were 74.0, 68.4 and 67.3% after 1, 2 and 3 years, respectively, and median LRC was not reached. Both OS and PFS were significantly reduced when antibiotics were administered during the course of (chemo)radiation: median OS was about 10 months longer for patients who did not receive antibiotic treatment during (chemo)radiation (36 versus 26 months, *p* < 0.05). 2 year OS reached 50.7% for patients treated with antibiotics during (chemo)radiation and 59.5% for patients who did not receive antibiotics. While 90 day mortality did not differ that much between patients receiving peritherapeutic antibiotics and patients who did not (14.2 vs 10.0%), Kaplan–Meier OS curves continuously diverged during the follow-up time (Fig. 1). While the median PFS amounted to 10 months for patients receiving antibiotic therapy, it ranged at 24 months for the remaining patients (*p* < 0.05). 2-year PFS was decreased by more than 10% in the group of patients who required antibiotic treatment during (chemo)radiation (36.8 vs 49.5%). There was also a trend towards reduced LRC in patients who received antibiotics during (chemo)radiation (*p* = 0.081).

**Fig. 2:**
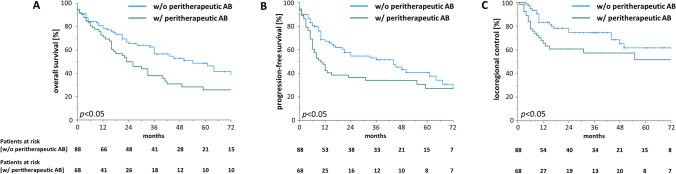
The negative prognostic role of antibiotic prescription is pronounced in patients aged 75 years and younger. Overall survival (**A**), progression-free survival (**B**) and locoregional control (**C**) of HNSCC patients aged < 75 years undergoing definitive radiotherapy (either alone or with concomitant systemic treatment) depending on peritherapeutic antibiotic administration. Groups were compared with log-rank tests. *AB* antibiotics, *w*/ with, *w/o* without

Remarkably, these findings were found to be more pronounced in patients below 75 years old (Fig. 2): Patients receiving antibiotics during (chemo)radiation in this age group exhibited a median OS of 26 months compared to 54 months for patients who did not (*p* < 0.05). Similarly, median PFS was considerably higher in patients who were not treated with antibiotics during treatment (44 vs 10 months, *p* = 0.05). In addition, LRC was significantly higher in the subgroup of patients who did not receive antibiotic treatment during the course of (chemo)radiation (2 year LRC 75.8% vs 60.4%, *p* < 0.05). In contrast, the oncological outcomes of the older and oldest old patients (≥ 75 years) were not influenced by peritherapeutic antibiotic treatment (*p* = 0.449 for OS, *p* = 0.788 for PFS, *p* = 0.911 for LRC) (Supplementary Fig. 1).

**Fig. 3:**
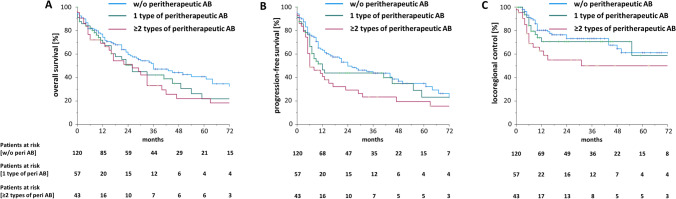
Higher numbers of applied types of antibiotics go along with reduced progression-free survival and locoregional control. Overall survival (**A**), progression-free survival (**B**) and locoregional control (**C**) of HNSCC patients treated with definitive radiotherapy (alone or with concomitant chemotherapy) in dependence of the applied types of antibiotics during radiotherapy. Groups were compared with log-rank tests. *AB* antibiotics, *peri* peritherapeutic, *w*/ with, *w/o* without

We observed a stronger reduction of both PFS and LRC in patients who were treated with two or more types of antibiotics during (chemo)radiation (*p* < 0.05 regarding the comparison between patients without antibiotic treatment and patients receiving ≥ 2 types of antibiotics, both for PFS and for LRC) (Fig. 3). There was a trend towards diminished OS in patients treated with ≥ 2 types of antibiotics when compared to patients not receiving any antibiotics during (chemo)radiation (*p* = 0.092).

As antibiotic treatment was found to be applied more often in patients with locoregionally advanced cancers, we also conducted a subgroup analysis in which only patients with UICC stage III-IV were analyzed (Supplementary Fig. 2). Similar to the whole cohort, both OS (*p* < 0.05) and PFS (*p* < 0.05) were significantly reduced in patients that were treated with antibiotics during the course of (chemo)radiation. In addition, there was a trend towards lower LRC rates in patients who received antibiotics during (chemo)radiation (*p* = 0.084).

In contrast to the prognostic role of peritherapeutic antibiotic application, antibiotic treatment within 30 days prior to start of (chemo)radiation (i.e., pretherapeutic) did not result in reduced OS (*p* = 0.348), PFS (*p* = 0.365) or LRC (*p* = 0.614) (Supplementary Fig. 3).

A further exploratory analysis was performed in which the cohort was separated into three groups: (A) Patients who did not receive antibiotics within the analyzed time interval (i.e., 30 days before (chemo)radiation until end of (chemo)radiation), (B) patients who only received prophylactic single-shot antibiotics, and (C) patients who were treated with antibiotics for other than prophylactic indication (patients who received both prophylactic single-shot antibiotics and antibiotics for another indication belonged to this group) (Supplementary Fig. 4). There was no significant difference between these three groups, neither regarding OS (*p* = 0.372), PFS (*p* = 0.363), or LRC (*p* = 0.605).

### Antibiotic application is an independent outcome prognosticator in patients aged < 75 years

We performed Cox proportional hazards regression analyses including patient- and tumor-related characteristics in order to examine whether peritherapeutic antibiotic application remained an independent prognosticator for survival. In the entire cohort, age (HR = 1.031, 95% CI 1.013–1.049, *p* = 0.001), ECOG (HR = 2.176, 95% CI 1.644–2.880, *p* < 0.001), T stage (HR = 1.315, 95% CI 1.872–1.613, *p* = 0.009) and concomitant systemic treatment (HR = 0.511, 95% CI 0.333–0.783, *p* = 0.002) were prognosticators for OS, while the prognostic value of peritherapeutic antibiotic administration (HR = 1.407, 95% CI 0.995–1.990, *p* = 0.053) reached borderline significance (Table 3). In the multivariate analysis, only ECOG (HR = 1.762, 95% CI 1.297–2.392, *p* < 0.001) and T stage (HR = 1.314, 95% CI 1.059–1.630, *p* = 0.013) were independent prognostic variables for OS, whereas administration of antibiotics during (chemo)radiation (HR = 1.319, 95% CI 0.930–1.871, *p* = 0.121) was not a statistically significant parameter.

**Table 3:** Uni- and multivariate Cox proportional hazards regression analysis of several patient- und tumor-related parameters in terms of overall survival and progression-free survival (*n* = 220)

Overall survival	Univariate			Multivariate		
	HR	95% CI	*p*	HR	95% CI	*p*
Age (continuous)	1.031	1.013–1.049	0.001	1.010	0.989–1.031	0.342
Gender (reference: female)	1.295	0.856–1.959	0.220			
ECOG (continuous)	2.176	1.644–2.880	< 0.001	1.762	1.297–2.392	< 0.001
T stage (continuous)	1.315	1.072–1.613	0.009	1.314	1.059–1.630	0.013
N stage (continuous)	1.100	0.880–1.375	0.402			
UICC (continuous)	1.106	0.854–1.431	0.445			
Tumor localization (reference: oropharynx)	0.827	0.585–1.168	0.280			
Tumor localization (reference: hypopharynx)	0.834	0.545–1.276	0.402			
Tumor localization (reference: larynx)	0.963	0.574–1.613	0.885			
Concomitant systemic treatment	0.511	0.333–0.783	0.002	0.628	0.362–1.091	0.099
Peritherapeutic antibiotics	1.407	0.995–1.990	0.053	1.319	0.930–1.871	0.121

Progression-free survival	Univariate			Multivariate		
	HR	95% CI	*p*	HR	95% CI	*p*
Age (continuous)	1.027	1.010–1.044	0.002	1.004	0.983–1.024	0.731
Gender (reference: female)	1.301	0.874–1.935	0.195			
ECOG (continuous)	1.918	1.465–2.511	< 0.001	1.563	1.161–2.105	0.003
T stage (continuous)	1.355	1.111–1.651	0.003	1.383	1.120–1.706	0.003
N stage (continuous)	1.162	0.938–1.438	0.169			
UICC (continuous)	1.123	0.885–1.424	0.340			
Tumor localization (reference: oropharynx)	0.878	0.626–1.231	0.450			
Tumor localization (reference: hypopharynx)	0.945	0.638–1.519	0.945			
Tumor localization (reference: larynx)	0.958	0.587–1.563	0.863			
Concomitant systemic treatment	0.562	0.374–0.844	0.006	0.583	0.339–1.001	0.051
Peritherapeutic antibiotics	1.397	0.995–1.962	0.053	1.327	0.941–1.872	0.106

*CI* confidence interval, *ECOG* Eastern Cooperative Oncology Group, *HR* Hazard ratio, *UICC* Union for International Cancer Control

Similar to the OS analyses, age (HR = 1.027, 95% CI 1.010–1.044, *p* = 0.002), ECOG (HR = 1.918, 95% CI 1.465–2.511, *p* < 0.001), T stage (HR = 1.355, 95% CI 1.111–1.651, *p* = 0.003) and systemic treatment (HR = 0.562, 95% CI 0.374–0.844, *p* = 0.006) were significant prognosticators for PFS, while there was a trend for peritherapeutic antibiotic administration (HR = 1.397, 95% CI 0.995–1.962, *p* = 0.053). Only ECOG (HR = 1.563, 95% CI 1.161–2.105, *p* = 0.003) and T stage (HR = 1.383, 95% CI 1.120–1.706, *p* = 0.003) remained prognostic parameters for PFS in the multivariate analysis.

We also performed a subgroup analysis of the patients aged < 75 years to exclude the older olds and oldest old patients according to the commonly used subclassification of elderly people [25, 26]. Here, besides ECOG performance status (HR = 2.610, 95% CI 1.774–3.840, *p* < 0.001), peritherapeutic antibiotic administration (HR = 1.703, 95% CI 1.106–2.623, *p* = 0.016) was the only independent parameter regarding OS (Table 4). Similarly, ECOG (HR = 2.263, 95% CI 1.560–3.284, *p* < 0.001), T stage (HR = 1.355, 95% CI 1.051–1.746, *p* = 0.019) and antibiotic administration during (chemo)radiation (HR = 1.550, 95% CI 1.009–2.380, *p* = 0.045) showed significant prognostic value concerning PFS in the cohort of HNSCC patients < 75 years.

**Table 4:** Uni- and multivariate Cox proportional hazards regression analysis of several patient- und tumor-related parameters in terms of overall survival and progression-free survival in the subgroup of patients younger than 75 years (*n* = 156)

Overall survival	Univariate			Multivariate		
	HR	95% CI	*p*	HR	95% CI	*p*
Age (continuous)	1.012	0.984–1.041	0.413			
Gender (reference: female)	1.260	0.756–2.100	0.375			
ECOG (continuous)	2.594	1.790–3.758	< 0.001	2.610	1.774–3.840	< 0.001
T stage (continuous)	1.267	0.988–1.626	0.063	1.251	0.968–1.616	0.088
N stage (continuous)	1.119	0.842–1.487	0.438			
UICC (continuous)	1.004	0.731–1.380	0.980			
Tumor localization (reference: oropharynx)	0.809	0.529–1.239	0.330			
Tumor localization (reference: hypopharynx)	0.806	0.483–1.345	0.409			
Tumor localization (reference: larynx)	0.875	0.448–1.771	0.697			
Concomitant systemic treatment	0.587	0.237–1.459	0.252			
Peritherapeutic antibiotics	1.635	1.066–2.508	0.024	1.703	1.106–2.623	0.016

Progression-free survival	Univariate			Multivariate		
	HR	95% CI	*p*	HR	95% CI	*p*
Age (continuous)	1.014	0.986–1.042	0.344			
Gender (reference: female)	1.183	0.723–1.934	0.504			
ECOG (continuous)	2.272	1.588–3.251	< 0.001	2.263	1.560–3.284	< 0.001
T stage (continuous)	1.342	1.049–1.717	0.019	1.355	1.051–1.746	0.019
N stage (continuous)	1.225	0.928–1.617	0.152			
UICC (continuous)	1.070	0.789–1.451	0.664			
Tumor localization (reference: oropharynx)	0.899	0.590–1.371	0.621			
Tumor localization (reference: hypopharynx)	0.976	0.576–1.652	0.927			
Tumor localization (reference: larynx)	0.903	0.477–1.711	0.755			
Concomitant systemic treatment	0.522	0.226–1.205	0.128			
Peritherapeutic antibiotics	1.521	0.995–2.325	0.053	1.550	1.009–2.380	0.045

*CI* confidence interval, *ECOG* Eastern Cooperative Oncology Group, *HR* Hazard ratio, *UICC* Union for International Cancer Control

As a further exploratory analysis, we analyzed a potential association between the duration of antibiotic treatment and survival (Supplementary Table 1). Even after excluding patients who only received single-shot antibiotics, there was no association between the duration of antibiotic treatment and OS or PFS.

## Discussion

In this large retrospective study of 220 HNSCC patients treated with definitive (chemo)radiation, we could demonstrate the negative prognostic value of peritherapeutic antibiotic administration during (chemo)radiation. We detected a stronger reduction of both PFS and LRC in patients who had received two or more types of antibiotics during (chemo)radiation. In contrast, the total duration of antibiotic treatment was not associated with the oncological outcomes. Additionally, to the best of our knowledge, we could show for the first time that unlike peritherapeutic antibiotic treatment, antibiotic administration prior to (chemo)radiation had no significant impact on the oncological outcomes. Antibiotic administration was found to be an independent prognosticator for survival in patients below 75 years, whereas we could not detect a prognostic effect within the cohort of patients ≥ 75 years.

So far, some preclinical and few small clinical studies have suggested that the gut microbiome can modulate the anti-tumor and normal tissue effects of radiotherapy [16, 27–30]. For instance, Crawford and Gordon could detect a radioprotective role of fasting-induced adipose factor, whose expression is suppressed by gut microbiota, on the intestinal epithelium [16]. Within a cohort of 45 rectal cancer patients, *Bacteroidales* were significantly less abundant in those patients that exhibited a complete response after chemoradiation, and *Duodenibacillus massiliensis* was also found associated with a complete response rate [28]. Shiao et al. demonstrated that intestinal fungi could regulate the antitumor immune response after irradiation in animal models of breast cancer and melanoma [29]. Whereas fungicide-induced fungal depletion (using 5-fluorocytosine or fluconazole) was found to enhance the anti-tumor efficacy of radiotherapy, antibiotic-mediated depletion (using ampicillin, imipenem, cilastatin, and vancomycin) of bacteria had the opposite effect. Mechanistically, it has been suggested that commensal bacteria may support the generation of activated T cells after irradiation, whereas commensal fungal species modulated the immunosuppressive tumor microenvironment by promoting pro-tumorigenic macrophages.

While in our cohort patients treated with peritherapeutic antibiotics more often had locoregionally advanced HNSCCs, causing an imbalance of tumor stages between the two groups, a subgroup analysis, in which only patients with locoregionally advanced HNSCCs (UICC III-IV) were included, revealed again inferior survival of patients exposed to antibiotics during treatment. Additionally, at least in the cohort of younger patients, peritherapeutic antibiotic treatment remained an independent variable for impaired survival also in the multivariate analysis. Considering the previous analysis of Nenclares et al. and our findings (taken together resulting in 492 analyzed patients), it is at least conceivable that antibiotic treatment during (chemo)radiation could be an independent prognostic factor in HNSCC patients.

Our study is the first that separately analyzed the impact of peritherapeutic and pretherapeutic antibiotic prescription on the outcomes of HNSCC patients receiving (chemo)radiation. This is different from the study of Nenclares et al., in which antibiotic administration was considered if applied within a time interval ranging from one week before the start of (chemo)radiation until two weeks after the end of (chemo)radiation [11]. In a recent meta-analysis, the negative prognostic effect of antibiotics in cancer patients receiving immune checkpoint inhibitors was present independently of the time of antibiotic administration in relation to immune checkpoint inhibitor treatment [19]. Another study with 360 non-small cell lung cancer (NSCLC) and renal cell carcinoma patients could show that the negative influence of antibiotics given 60 days prior to immune checkpoint inhibitors was not as strong as within the first 30 days before immune checkpoint inhibitor therapy [31]. To what extent recovery of the antibiotic-induced dysregulated microbiome may explain the differences between pre- and peritherapeutic antibiotic administration observed in our study is unknown and can only be explored by repeated microbiome analyses after antibiotic treatment and during (chemo)radiation.

Furthermore, we could show that the detrimental effects of antibiotics mostly affect younger patients (< 75 years) and are absent in very old patients (≥ 75 years). Presence of immunosenescence, resulting in impaired anti-tumor capacities of the immune system, and a higher proportion of patients with dysbiosis (independently of antibiotic treatment) in the elderly population could be potential explanations [24, 32]. As antibiotics have also shown to negatively interfere with the anti-tumor efficacy of chemotherapeutic agents and to impair survival rates after chemotherapy [33, 34], the lower usage of concomitant chemotherapy in patients aged 75 years and older may also contribute to the absent prognostic role of peritherapeutic antibiotic treatment.

Despite our findings on the adverse role of peritherapeutic antibiotics in HNSCC patients, antibiotic therapies are often necessary to treat life-threating complications during (chemo)radiation including pneumonia, sepsis and port catheter or gastric feeding tube infections. Nevertheless, caution may be warranted when prescribing antibiotics in this population, and empirical antibiotic treatments, e.g., based on laboratory results such as C-reactive protein serum levels, may be performed more reservedly. In this context, the risk for severe infection-related complications, for which HNSCC patients often exhibit risk factors such as lymphopenia and malnutrition, must be critically weighted against the potential negative microbiome alterations caused by antibiotics [35]. In the future, (tailored) administration of probiotics or fecal microbiome transfers may be other approaches to address antibiotics-induced microbiome alterations; however, so far, these approaches are mainly examined with the aim to alleviate radiotherapy-induced normal tissue toxicities but not to increase the anti-tumor efficacy of radiotherapy (reviewed in [21] and [30]). At least for immune checkpoint inhibitor treatment, there are preclinical and early clinical studies showing the potential of those approaches to increase the anti-tumor immune response [13, 36, 37]

Although there is no evidence yet for a benefit in combining immune checkpoint blockade with definitive chemoradiation in HNSCC, the relevance of peritherapeutic antibiotic administration may increase in clinical scenarios in which immune checkpoint inhibitors would be administered as consolidative treatment after definitive chemoradiation, as it was performed in the PACIFIC trial in NSCLC and as it is currently evaluated in several studies such as the IMvoke010 (NCT03452137) or EA3161 (NCT03811015) trials for HNSCC [38, 39]. At least for metastatic cancer patients, there now is increasing cumulative evidence that antibiotic therapy prior to or parallel with immune checkpoint inhibitor treatment results in impaired oncological outcomes [40, 41]. As shown in the meta-analysis of Tsikala-Vafea, both OS (adjusted HR = 1.87) and PFS (adjusted HR = 1.93) were shorter, while response rates (odds ratio = 0.54) after immune checkpoint inhibitor therapy were lower in patients exposed to antibiotics [40]. However, these findings predominantly derive from NSCLC, renal cell carcinoma and melanoma, and there are only few studies (presented as conference abstracts or studies with mixed cohorts including several cancer types) that evaluated the prognostic role of antibiotics in recurrent and/or metastatic HNSCC patients undergoing immune checkpoint inhibitor therapy [42–44].

In addition to the microbiome-influencing abilities of antibiotics, their potential anti-tumor and radiosensitizing effects of antibiotics must be considered. For instance, doxycycline (which was used in only one case in our cohort) was found to radiosensitize cancer stem-like cells by a factor of 4.5 in vitro [22, 30]. Other antibiotics that were reported to enhance the anti-tumor effects of radiotherapy are cephalosporins [23], which were the most common antibiotic type in our cohort. However, as the majority of cephalosporin courses were applied in patients receiving single-shot prophylactic antibiotic treatment for port catheter insertion, these potentially radiosensitizing effects are probably insignificant given the serum half-time between 1 and 2 h of most cephalosporins [45] and the fact that port catheter insertion often was performed prior to the start of radiotherapy treatment.

There are several limitations of our study that are mainly related to the retrospective nature of the analysis. Compared to the study of Nenclares and colleagues, our cohort comprised of a considerably older patient population, resulting in lower survival rates and thus complicating direct comparisons between these studies. The sample size did not allow to separately investigate the different classes of antibiotics, which however would be an important point to be addressed in future multicenter cohort studies. For instance, vancomycin has been found to potentiate the radiotherapy-induced antitumor immune response by eliminating butyrate-producing bacteria in preclinical models [27]. Due to the low number of patients receiving oral antibiotics (*n* = 18 courses with oral antibiotics), we did not perform a subgroup analysis comparing the effects between intravenous and oral antibiotics, which would be an interesting point for further larger analyses on this topic. We also could not examine the different subsites of the head-and-neck region, although it is known that the microbiome differs for example between oral cavity and oropharyngeal carcinoma patients [46]. Our correlative analyses concerning the prognostic role of antibiotic administration during (chemo)radiation cannot prove a causative effect of antibiotics in this context, as also the underlying diseases requiring antibiotic treatment (e.g., pneumonia, port catheter infection, urogenital tract infection, sepsis) can worsen the outcomes of HNSCC patients after (chemo)radiation. We could at least exclude differences regarding patient age, ECOG status, radiotherapy discontinuation rate, total radiotherapy dose and total radiotherapy duration in patients requiring antibiotic treatment. The Kaplan–Meier survival curves that increasingly diverged over the follow-up period also indicate that the negative prognostic role of peritherapeutic antibiotic therapy was not solely related to an increase in early (treatment-related) mortality. Due to the time period in which patients were treated, routine HPV testing was not performed for a substantial number of oropharyngeal carcinoma patients, ruling out further analyses of this parameter in our study.

Given these limitations, our study should be considered as a hypothesis-generating analysis pointing out the potential prognostic relevance of antibiotic administration during (chemo)radiation in HNSCC patients and providing a clinical basis for mechanistical analyses on this topic. Consequently, some few studies are currently investigating the influence of patients’ microbiome on the anti-tumor efficacy of radiotherapy in HNSCC patients (e.g., COMRAD-HNSCC [NCT05156177] and [47]).

## Conclusion

Peritherapeutic antibiotic usage was associated with impaired oncological outcomes of HNSCC patients undergoing definitive (chemo)radiation, whereas pretherapeutic exposure to antibiotics was not associated with diminished survival. The adverse prognostic value was pronounced in patients younger than 75 years, in whom peritherapeutic antibiotic administration was an independent adverse prognostic parameter. Further prospective and multicenter studies with larger sample sizes and including repeated microbiome analyses are required to validate our observations and to elucidate the mechanisms underlying the interplay between antibiotic administration, microbiome alterations and patient outcomes in HNSCC.

## Supplementary Information

Below is the link to the electronic supplementary material.

Supplementary file1 (TIF 632 KB)

Supplementary file2 (TIF 700 KB)

Supplementary file3 (TIF 716 KB)

Supplementary file4 (TIF 131 KB)

Supplementary file5 (DOCX 14 KB)

## Data Availability

The data that support the findings of this study are available on reasonable request from the corresponding author.
