# Supplementary figures and images for: The influence of antibiotic administration on the outcomes of head-and-neck squamous cell carcinoma patients undergoing definitive (chemo)radiation
Source: Eur Arch Otorhinolaryngol. 2023 Feb 10;280(5):2605–16. doi: 10.1007/s00405-023-07868-3 (PMC10066162; doi:10.1007/s00405-023-07868-3)

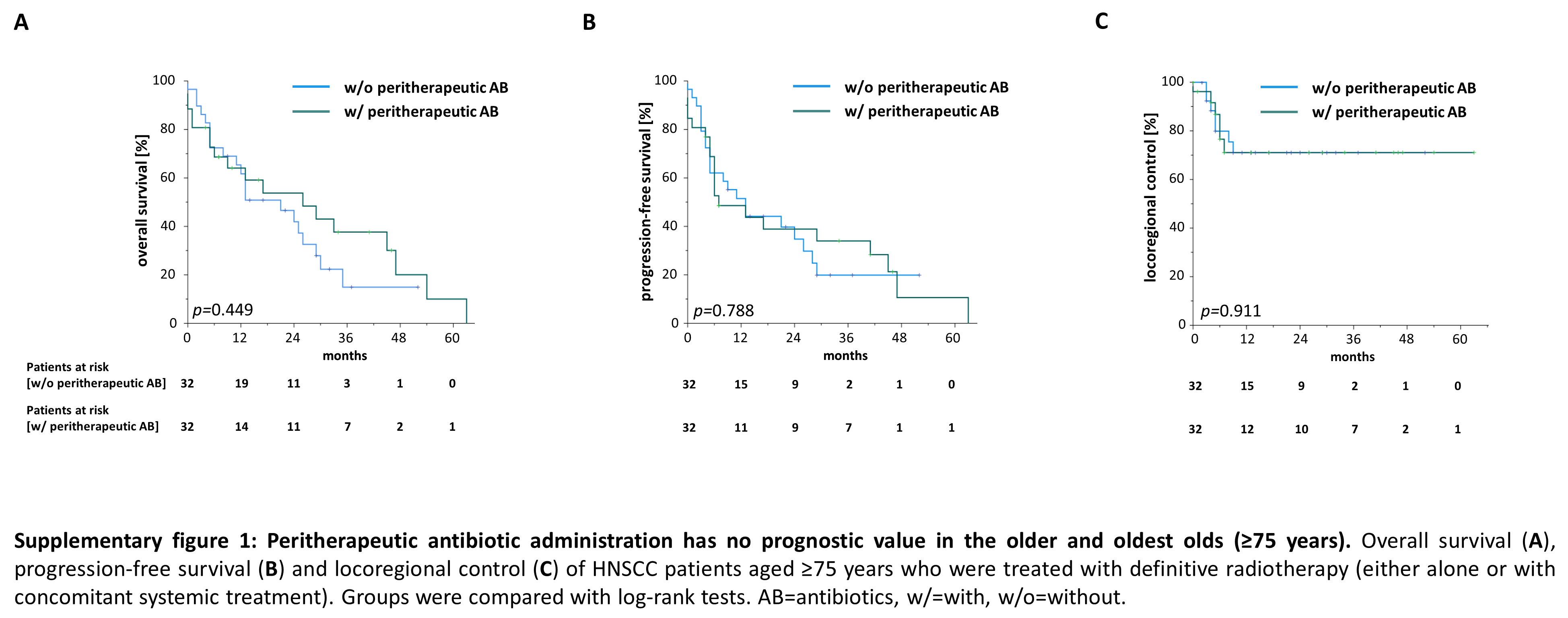

Supplement: Supplementary file 1 — Supplementary file1 (TIF 632 KB) [file 405_2023_7868_MOESM1_ESM.tif]

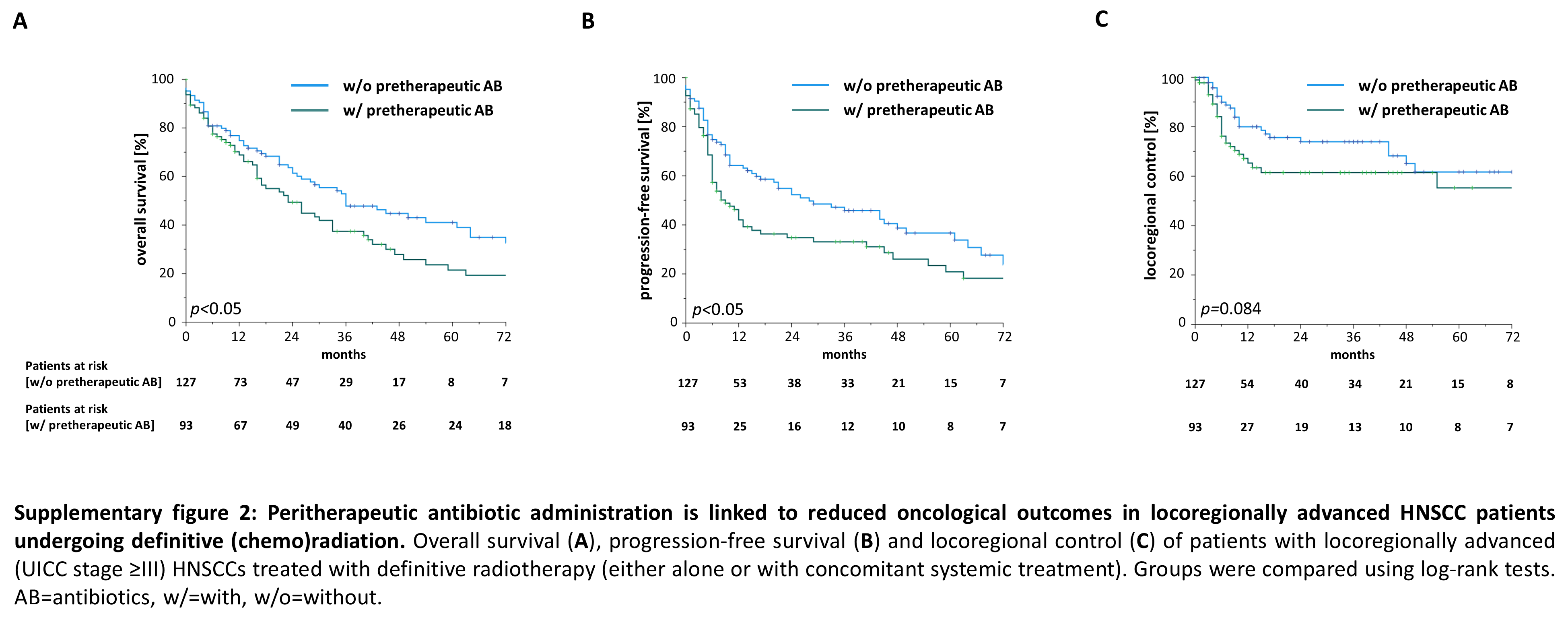

Supplement: Supplementary file 2 — Supplementary file2 (TIF 700 KB) [file 405_2023_7868_MOESM2_ESM.tif]

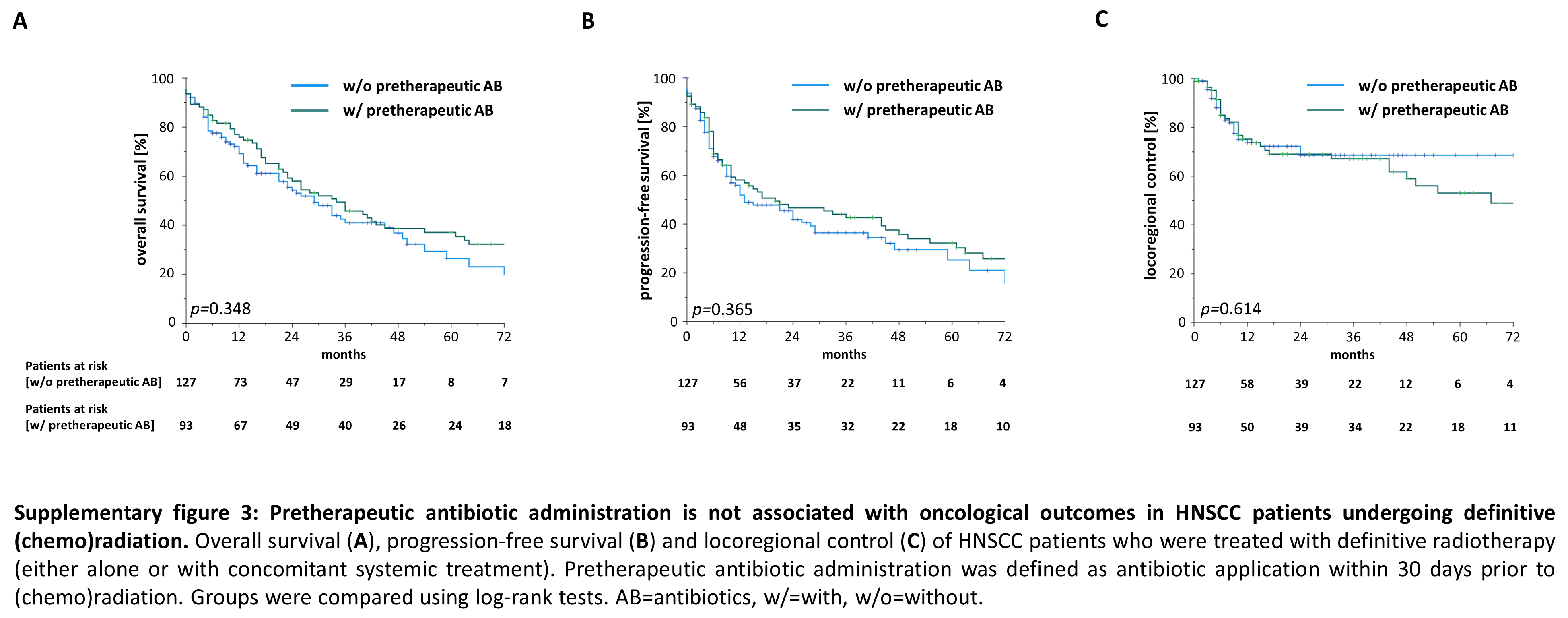

Supplement: Supplementary file 3 — Supplementary file3 (TIF 716 KB) [file 405_2023_7868_MOESM3_ESM.tif]

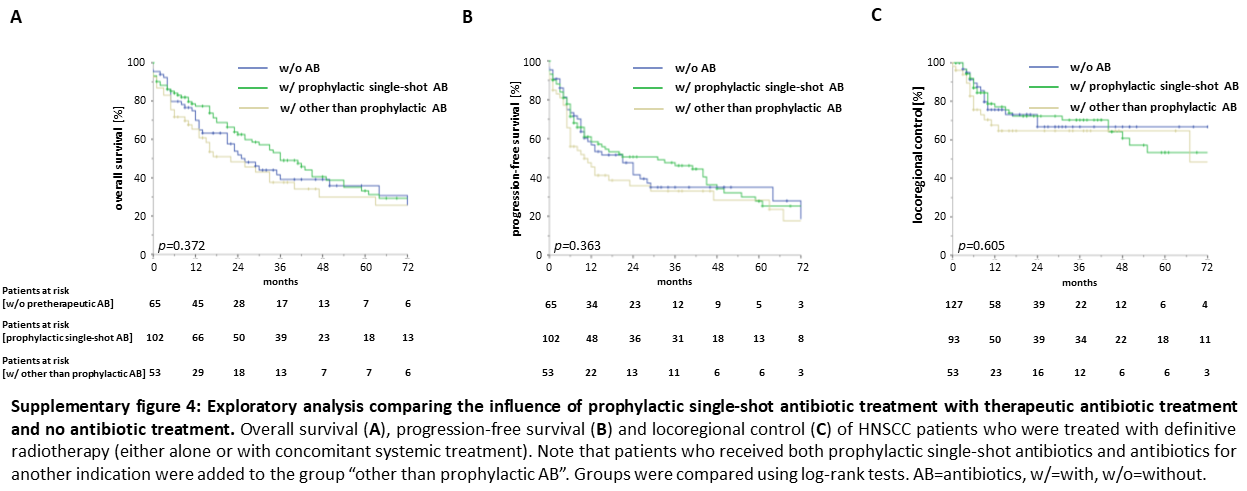

Supplement: Supplementary file 4 — Supplementary file4 (TIF 131 KB) [file 405_2023_7868_MOESM4_ESM.tif]
