# Supplementary material for: The influence of antibiotic administration on the outcomes of head-and-neck squamous cell carcinoma patients undergoing definitive (chemo)radiation
Source: Eur Arch Otorhinolaryngol. 2023 Feb 10;280(5):2605–16. doi: 10.1007/s00405-023-07868-3 (PMC10066162; doi:10.1007/s00405-023-07868-3)
Supplement: Supplementary file 5 — Supplementary file5 (DOCX 14 KB) [file 405_2023_7868_MOESM5_ESM.docx]

**Supplementary table 1: Univariate Cox proportional hazards regression analysis regarding the association between total duration of antibiotic treatment (per patient) with overall survival and progression-free survival.** CI=Confidence interval, HR=Hazard ratio.

| **Overall survival** |  |  |  |
| --- | --- | --- | --- |
|  | **HR** | **95% CI** | ***p*** |
| Total duration of antibiotic treatment (continuous) | 1.005 | 0.986-1.025 | 0.605 |
| Total duration of antibiotic treatment excluding single-shot antibiotics (continuous) | 0.989 | 0.960-1.019 | 0.469 |
| **Progression-free survival** |  |  |  |
|  | **HR** | **95% CI** | ***p*** |
| Total duration of antibiotic treatment (continuous) | 1.001 | 0.982-1.021 | 0.904 |
| Total duration of antibiotic treatment excluding single-shot antibiotics (continuous) | 0.982 | 0.952-1.012 | 0.237 |
